# Levels of Systemic Low-grade Inflammation in Pregnant Mothers and Their Offspring are Correlated

**DOI:** 10.1038/s41598-019-39620-5

**Published:** 2019-02-28

**Authors:** Nadia Rahman Fink, Bo Chawes, Klaus Bønnelykke, Jonathan Thorsen, Jakob Stokholm, Morten Arendt Rasmussen, Susanne Brix, Hans Bisgaard

**Affiliations:** 1 COPSAC, Copenhagen Prospective Studies on Asthma in Childhood, Herlev and Gentofte Hospital, University of Copenhagen, Copenhagen, Denmark; 2 Department of Pediatrics, Naestved Hospital, Naestved, Denmark; 3 Department of Food Science, Faculty of Science, University of Copenhagen, Copenhagen, Denmark; 4 Department of Biotechnology and Biomedicine, Technical University of Denmark, Lyngby, Denmark

## Abstract

High sensitivity C-reactive protein (hs-CRP) is a marker of systemic low-grade inflammation and associated with chronic inflammatory diseases. It is unknown whether maternal and infant hs-CRP levels are correlated and little is known about risk factors in early childhood. Hs-CRP were measured in mothers during pregnancy week 24 (N = 690), and one-week postpartum (N = 675) and in their children age 6 mo (N = 640) enrolled in the Copenhagen Prospective Studies on Asthma in Childhood_2010_ (COPSAC_2010_) cohort. The risk factor analysis included anthropometrics, environmental exposures and CRP-Genetic Risk Score (GRS). Mother’s body mass index (BMI), use of antibiotics, smoking, cesarean delivery and season were associated with higher maternal hs-CRP level, whereas higher social circumstances were associated with lower hs-CRP level (p < 0.05). Child’s BMI, siblings, bacterial airway colonization, current infection, CRP-genetic risk score and season were associated with higher hs-CRP at age 6 mo (all p < 0.05). Mother’s hs-CRP level in pregnancy week 24 was associated with hs-CRP level in the child at 6 mo: β-coefficient = 0.11 [95% CI: 0.01–0.20], R^2^ = 0.22, p = 0.03. The association was unchanged adjusted for all significant risk factors. Systemic low-grade inflammation in pregnant mothers and their offspring is correlated independently of BMI, environmental exposures and genetic risk factors.

## Introduction

C-reactive protein (CRP) level has been reported to be elevated in pregnant women without pregnancy complications as compared to non-pregnant women^1,2^. The degree of this low-grade systemic inflammation in pregnancy has been associated with environmental exposures including smoking and diet^3,4^, and with conditions such as preeclampsia, obesity and gestational diabetes, which are all well-known fetal stressors that can cause developmental adaptions^5–8^. Chronic low-grade inflammation in pregnancy is suspected to be potentially harmful for the developing fetus^9^, and may initiate a trajectory towards immune diseases such as asthma and allergy in childhood^10^.

There is a paucity of studies investigating risk factors for systemic low-grade inflammation in early life, but we and other groups have previously linked hs-CRP levels to neonatal lung function^11^, allergic sensitization^12^, and childhood obesity^4^, which may in turn affect the child’s risk of developing other chronic inflammatory disorders later in life^13^.

We hypothesized that systemic low-grade inflammation in pregnant women may influence the child’s risk of such inflammation. To investigate this link, we examined the association between levels of hs-CRP measured in mother-child pairs participating in the Copenhagen Prospective Studies on Asthma in Childhood_2010_ (COPSAC_2010_) cohort adjusting for anthropometrics, genetics and environmental confounders.

## Methods

### Study population

The participants were from the ongoing clinical COPSAC_2010_ population-based mother-child cohort^14^. A total of 738 pregnant women were recruited for the COPSAC_2010_ cohort, whereof 38 withdrew before giving birth. There were five twin pairs in the cohort; only one child of each twin-pair was used in the analysis to avoid duplicating mothers’ data, leaving 695 mother-child pairs in this study. Further detail on recruitment, inclusion and exclusion criteria were previously described in detail^15^.

### Ethics

The study was conducted in accordance with the guiding principles of the Declaration of Helsinki and was approved by the National Committee on Health Research Ethics (H-B-2008-093) and the Danish Data Protection Agency (2015-41-3696). Both parents gave oral and written informed consent before enrolment.

### Assessment of hs-CRP levels

Blood was drawn from a cubital vein into an EDTA tube from women at pregnancy week 24 and one-week postpartum and from children at age 6 months, centrifuged to separate plasma and cells, and immediately stored at −80 °C until analysis. After thawing of samples, hs-CRP levels were determined by a high sensitivity electrochemiluminescence-based assay from MesoScale Discovery. Samples were measured in duplicate and read by using the Sector Image 2400 A (Meso Scale Discovery, Gaitherburg, MD). The lower limit of detection of CRP was 0.007 ng/mL.

### Risk factors

All data were retrieved and entered in a dedicated online database at clinical interviews with the parents at the COPSAC research unit using predefined questions with closed response categories. The families visited the research unit at week 24 and 36 of pregnancy, one-week postpartum and subsequently at child age 1 week, 1, 3 and 6 months.

We collected information regarding mothers’ doctor-diagnosed asthma, allergy or eczema (yes/no), smoking during pregnancy (yes/no), antibiotic use during pregnancy prior to mothers’ blood sampling (yes/no), parity/prior birth (yes/no) and preeclampsia (yes/no). Mothers’ body mass index (BMI) was calculated based on pre-pregnancy weight and height collected from the participant pregnancy charts and matched with data from the Danish Fetal Database. Data on pre-pregnancy BMI, parity, preeclampsia and antibiotic use in pregnancy were validated against national register data.

Socioeconomic status was represented as the z-score of principle component 1 from a principle component analysis including household income, mothers’ age and level of education.

The child’s length and weight were measured at the COPSAC clinic at age 6 months. Age- and gender specific BMI z-scores were subsequently calculated using WHO standardized macros. Exclusive breastfeeding for more than 4 months (yes/no), daycare start before age 6 months (yes/no), siblings at home (yes/no), cat (yes/no) and dog (yes/no) in the home were registered. Bacterial colonization of the airway with *M. catharralis, H. influenzae* and/or *S. pneumoniae* (yes/no) at age 4 weeks was determined as previously detailed^16^.

Current infection in a period of two weeks prior to the children’s blood sampling was determined by diary reported symptoms for more than one day of upper or lower respiratory tract infections, ear or throat pain, gastroenteritis or fever with unknown cause (yes/no).

The child’s CRP-genetic risk score (GRS) was calculated based on 17 single nucleotide polymorphisms (SNPs), selected based on a meta-analysis of genome-wide association studies (GWAS)^17^. The final score was based on the sum of risk alleles weighted on their reported effect size on CRP levels^17^ and subsequently z-score transformed.

Season of blood sampling was analyzed based on the day in year of sampling, day 1 to 365. Due to sinusoid distribution of data, it was transformed using a function taking such distribution into account (see Supplementary Methods for details).

### Statistical analysis

Levels of hs-CRP were log-transformed prior to analysis. Before log-transformation, the lower limit of detection was added to all completed measurements as a pseudo-count to avoid zero values.

Baseline characteristics of included and excluded children were analyzed by chi square test for categorical variables and by t-test for continuous variables. We analyzed risk factor associations with level of hs-CRP at each time-point separately (mothers’ level at pregnancy week 24, one-week postpartum, and child age 6 months) by using univariate linear models. Thereafter, we analyzed the associations between levels of hs-CRP at the three time-points using multivariable linear regression models adjusting for all significant risk factors (p < 0.05). Goodness of model fit was evaluated by marginal R squared values.

Finally, we conducted an analysis of the associations between levels of hs-CRP at the three time-points using a multivariable linear regression model with all risk factors included using a backward selection procedure eliminating risk factors stepwise until only significant risk factors (p < 0.05) were retained in the model.

All statistical analysis were done using the statistical software program ‘R’ version 3.2.3 (The R Foundation for Statistical Computing) and visualized with the “ggplot2” package.

### Governance

We are aware of and comply with recognized codes of good research practice, including the Danish Code of Conduct for Research Integrity. We comply with national and international rules on the safety and rights of patients and healthy subjects, including Good Clinical Practice (GCP) as defined in the EU’s Directive on Good Clinical Practice, the International Conference on Harmonisation’s (ICH) good clinical practice guidelines and the Helsinki Declaration. We follow national and international rules on the processing of personal data, including the Danish Act on Processing of Personal Data and the practice of the Danish Data Inspectorate.

## Results

### Baseline characteristics

**Figure 1:**
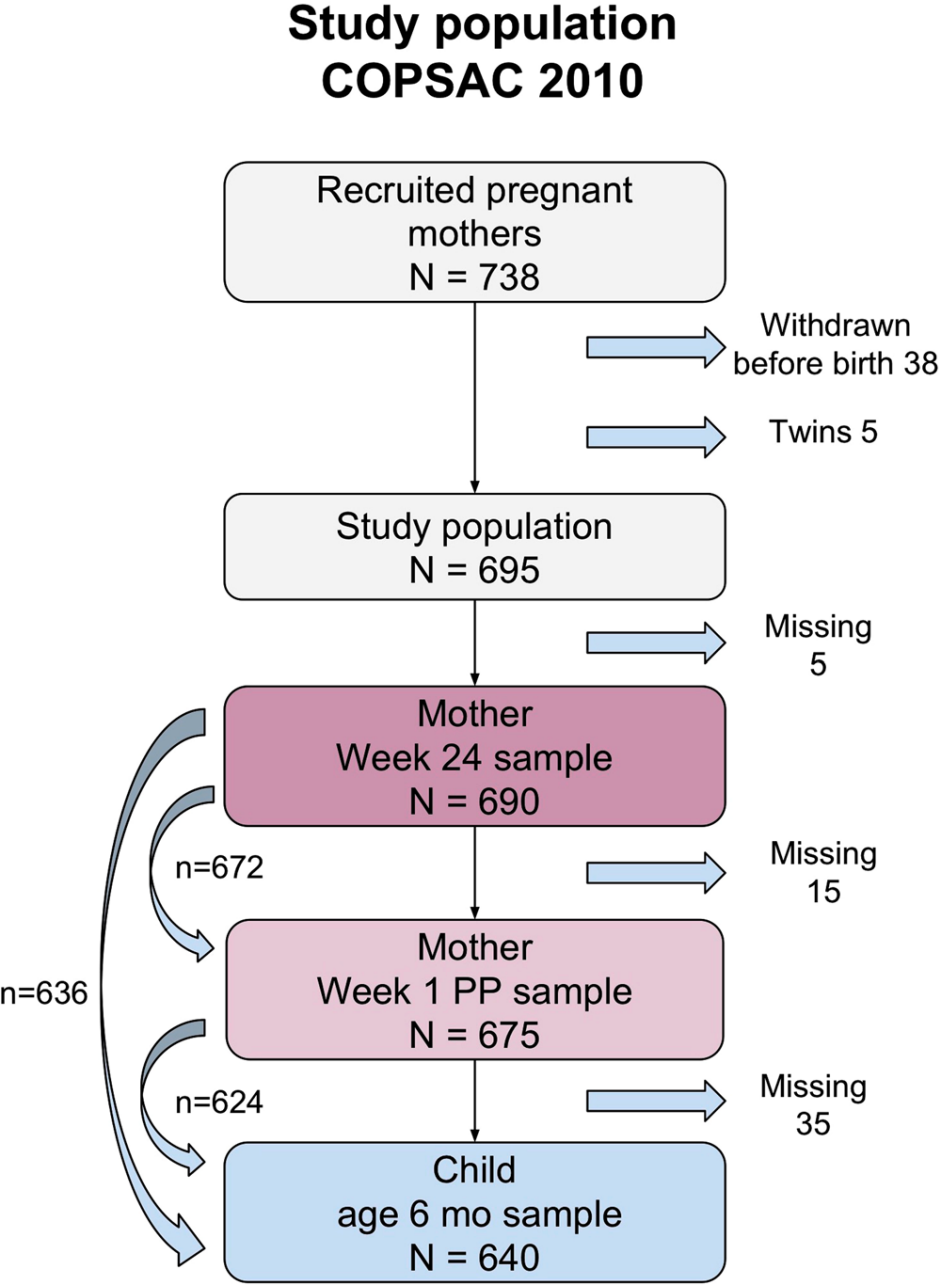
Flowchart of included mothers and children.

Blood samples were collected from 690 (99%) of the mothers at pregnancy week 24 and from 675 (97%) at one-week postpartum. At age 6 months, blood was collected from 640 (92%) of the children. A total of 622 (90%) mother-child pairs had blood available from all three time-points (Fig. 1). Baseline characteristics for included and excluded participants (with samples at all three time points) are represented in Table 1, showing no significant differences.

**Table 1:** Baseline characteristics of the study population.

	Included N = 622*	Excluded N = 73*	p-value
Current Infection, N (%)	170 (27.3)	5 (18.5)	0.21
Mother BMI, mean (Standard Deviation (SD))	24.56 (4.47)	24.49 (3.75)	0.91
Preeclampsia, N (%)	25 (4.0)	7 (9.6)	0.06
Antibiotic use in pregnancy, N (%)	229 (36.9)	24 (32.9)	0.59
Cesarean section (Delivery), N (%)	129 (20.7)	21 (28.8)	0.15
Male sex, N (%)	315 (51.1)	34 (49.3)	0.88
Child BMI, mean z-score (SD)	0.06 (0.91)	0.22 (1.05)	0.15
Parity, prior birth > 0, N (%)	339 (54.5)	37 (50.7)	0.62
Social circumstances, mean z-score (SD)	−0.00 (1.00)	−0.02 (1.01)	0.91
Smoking in pregnancy, N (%)	44 (7.1)	10 (13.7)	0.08
Cat in household in pregnancy, N (%)	129 (20.7)	16 (21.9)	0.94
Dog in household in pregnancy, N (%)	122 (19.6)	19 (26.0)	0.26
Cat in household in the 1^st^ year, N (%)	137 (22.3)	14 (20.6)	0.87
Dog in household in the 1^st^ year, N (%)	131 (21.3)	19 (27.9)	0.27
Fathers with asthma, allergy or eczema, N (%)	263 (43.5)	29 (40.8)	0.77
Mothers with asthma, allergy or eczema, N (%)	330 (53.1)	43 (58.9)	0.42
Neonatal bacterial airway colonization, N (%)	177 (29.4)	15 (23.1)	0.36
CRP-genetic risk score, mean z-score (SD)	0.01 (0.99)	−0.14 (1.09)	0.33
Passive smoking, N (%)	88 (14.3)	13 (19.1)	0.38
Daycare start before 6 months, N (%)	23 (3.7)	3 (4.4)	1.00
Solely breastfed for more than 4 months, N (%)	0.54 (0.50)	0.50 (0.50)	0.54
Older siblings at home, N (%)	357 (57.4)	37 (50.7)	0.33

*Population including all CRP measurements from mothers’ week 24, week-one postpartum and children age 6 months. Note, that due to missing data for the distinct exposure variable, sample size may vary.

### Risk factors of CRP levels in mother and child

Pregnancy week 24 included 690 samples, with hs-CRP Geometric Mean (GM) value = 5.70 mg/L and none below detection limit. Antibiotic use before pregnancy week 24, was associated with higher level of hs-CRP: GM, 6.75 vs. 5.41 mg/L; geometric mean ratio (GMR), 1.25 [95% Confidence Interval (CI): 1.03–1.50], p = 0.02. Women who smoked during pregnancy had higher hs-CRP: GM, 8.05 vs. 5.54 mg/L; GMR, 1.45 [95% CI: 1.07–1.97], p = 0.02, which was also the case for women with a pre-pregnancy BMI above 25 kg/m2: GM, 8.28 vs. 4.64 mg/L; GMR, 1.09 [95% CI: 1.07–1.11], p < 0.01. Social circumstances, z-score above 0, was negatively associated with hs-CRP: GM, 5.26 vs. 6.16 mg/L; GMR, 0.90 [95% CI: 0.83–0.98], p = 0.02, i.e. related to mothers education (higher), age (higher) and income level (higher). Moreover, season of sampling was significantly associated with the level of hs-CRP (p < 0.01), with higher levels of hs-CRP in winter compared to summer.

Mothers’ asthma, allergy or eczema, cat or dog in the household, preeclampsia and number of previous full born pregnancies (parity) were not associated with hs-CRP levels (Table 2 and Supplement Fig. 1a).

**Table 2:** Risk factor analysis of CRP levels at pregnancy week 24 (N = 690), one-week postpartum (N = 675) and children age 6 months (N = 640). GM = geometric mean, GMR = geometric mean ratio.

	Yes (N), GM, mg/L	No (N), GM, mg/L	GMR	95% CI	p-value
**Pregnancy week 24 (N** = **690)**					
Antibiotics during pregnancy	(173) 6.75	(516) 5.41	1.25	[1.03–1.50]	0.02
Mothers with asthma, allergy or eczema	(368) 5.53	(321) 5.92	0.93	[0.79–1.10]	0.43
Cat in household during pregnancy	(143) 6.05	(547) 5.62	1.08	[0.88–1.32]	0.47
Dog in household during pregnancy	(140) 6.04	(550) 5.62	1.08	[0.88–1.32]	0.50
Preeclampsia	(31) 7.50	(659) 5.63	1.33	[0.90–1.98]	0.14
Smoking in pregnancy	(54) 8.05	(636) 5.54	1.45	[1.07–1.97]	0.02
Mothers’ BMI, per BMI point*			1.09	[1.07–1.11]	<0.01
Mothers’ BMI > 25 kg/m^2^**	(245) 8.28	(440) 4.64			
Parity, multipara	(374) 5.68	(316) 5.73	0.99	[0.84–1.17]	0.92
Social-circumstances, per z-score point*			0.90	[0.83–0.98]	0.02
Social-circumstances, z-score > 0**	(337) 5.26	(353) 6.16			
Season of sampling	—	—	—	—	<0.01
**One-week postpartum (N = 675)**					
Antibiotics during pregnancy	(248) 18.12	(426) 16.17	1.12	[0.91–1.38]	0.28
Mothers with asthma, allergy or eczema	(360) 16.47	(314) 17.50	0.94	[0.77–1.15]	0.55
Cat in household during pregnancy	(141) 17.67	(534) 16.73	1.06	[0.83–1.36]	0.66
Dog in household during pregnancy	(135) 15.65	(540) 17.25	0.91	[0.71–1.17]	0.45
Preeclampsia	(29) 17.70	(646) 16.88	1.05	[0.64–1.72]	0.85
Smoking in pregnancy	(49) 18.61	(626) 16.79	1.11	[0.75–1.63]	0.60
Mother’s BMI, per BMI point*			1.03	[1.00–1.05]	0.02
Mother’s BMI > 25 kg/m^2^**	(236) 19.90	(435) 15.65			
Parity, multipara	(367) 17.12	(308) 16.68	1.03	[0.84–1.26]	0.78
Cesarean section	(143) 21.17	(532) 15.93	1.33	[1.04–1.70]	0.02
Social-circumstances, per z-score point*			0.93	[0.84–1.03]	0.16
Social-circumstances z-score > 0**	(331) 15.77	(344) 18.10			
Season of sampling	—	—	—	—	<0.01
**Child age 6 months (N = 640)**					
Neonatal bacterial airway colonization	(182) 0.18	(436) 0.12	1.53	[1.24–1.87]	<0.01
Older siblings at home	(365) 0.18	(275) 0.10	1.85	[1.55–2.22]	<0.01
Mothers with asthma, allergy or eczema	(342) 0.13	(297) 0.15	0.88	[0.73–1.06]	0.16
Cat in household in the 1st year	(141) 0.12	(492) 0.14	0.83	[0.67–1.04]	0.11
Dog in household in the 1st year	(137) 0.13	(496) 0.14	0.95	[0.75–1.19]	0.57
Current infection	(173) 0.22	(375) 0.11	2.08	[1.70–2.54]	<0.01
Daycare start before age 6 months	(24) 0.15	(610) 0.14	1.11	[0.68–1.81]	0.67
Passive smoking	(90) 0.12	(543) 0.14	0.86	[0.66–1.12]	0.32
Cesarean section	(137) 0.12	(503) 0.14	0.84	[0.68–1.07]	0.16
Sex, male	(326) 0.13	(309) 0.14	0.89	[0.74–1.07]	0.23
Solely breastfed > 4 months	(346) 0.15	(292) 0.12	1.18	[0.98–1.43]	0.08
BMI at 6 months, per z-score point*			1.11	[1.00–1.23]	0.04
BMI at 6 months, z-score > 0**	(319) 0.15	(316) 0.13			
CRP-genetic risk score, per z-score point*			1.27	[1.15–1.40]	<0.01
CRP-genetic risk score, z-score > 0**	(304) 0.17	(278) 0.11			
Social circumstances, per z-score point*			1.03	[0.93–1.12]	0.49
Social circumstances, z-score > 0**	(309) 0.15	(331) 0.13			
Season of sampling	—	—	—	—	<0.01

*GMR based on continuous variable. Per z-score point.

**Dichotomized variable to evaluate GM(mg/L) in two groups.

One-week postpartum included 675 samples, with hs-CRP GM = 16.91 mg/L, and none below detection limit. One week postpartum levels were up to 3-fold higher than at week 24 of pregnancy. Delivery mode was associated with higher hs-CRP: cesarean section vs. vaginal delivery, GM, 21.17 vs. 15.93 mg/L; GMR 1.33 [95% CI: 1.04–1.70], p = 0.02, likewise for pre-pregnancy BMI above 25 kg/m^2^: GM, 19.90 vs 15.65 mg/L; GMR, 1.03 [95% CI: 1.00–1.05], p = 0.02, and season of sampling, with higher levels in summer compared to winter, p < 0.01.

Antibiotic use during pregnancy, mothers with asthma, allergy or eczema, cat or dog in the household, preeclampsia, parity and social circumstances were not associated with the level of hs-CRP (Table 2 and Supplement Fig. 1b).

Child age 6 months included 640 samples, with GM = 0.14 mg/L, and none below detection limit. Living with at least one older sibling was significantly associated with higher hs-CRP: GM, 0.18 vs. 0.10 mg/L; GMR, 1.85 [95% CI: 1.55–2.22], p < 0.01, similarly for neonatal bacterial airway colonization at week 4: GM, 0.18 vs. 0.12 mg/L; GMR, 1.53 [95% CI: 1.24–1.87], p < 0.01, BMI z-score above 0: GM, 0.15 vs. 0.13 mg/L; GMR, 1.11 per BMI point [95% CI: 1.00–1.23], p = 0.04, and season of sampling, with higher levels in winter compared to summer, p < 0.01. A CRP-Genetic risk score (z-score) above 0 was also associated with higher hs-CRP: GM, 0.17 vs. 0.11 mg/L; GMR, 1.27, [95%CI: 1.15–1.40], p < 0.01, and likewise for having an infection 14 days prior to blood sampling: GM, 0.22 vs. 0.11 mg/L; GMR, 2.08 [95% CI: 1.70–2.54], p < 0.01.

Mothers with asthma, allergy or eczema, living with cat or dog, daycare start before age 6 months, exposure to passive smoking, delivery by cesarean section, male sex, exclusive breastfeeding for more than 4 months and social circumstances were not associated with the hs-CRP at 6 months (Table 2 and Supplement Fig. 1c).

### Association between hs-CRP levels during pregnancy and from mother to child

To examine the association between maternal hs-CRP levels in pregnancy and one-week post-partum as well as between mother and child, we performed a multivariable analysis adjusting for all significant risk factors (p < 0.05) from both of the two time points being compared (Table 2).

**Figure 2:**
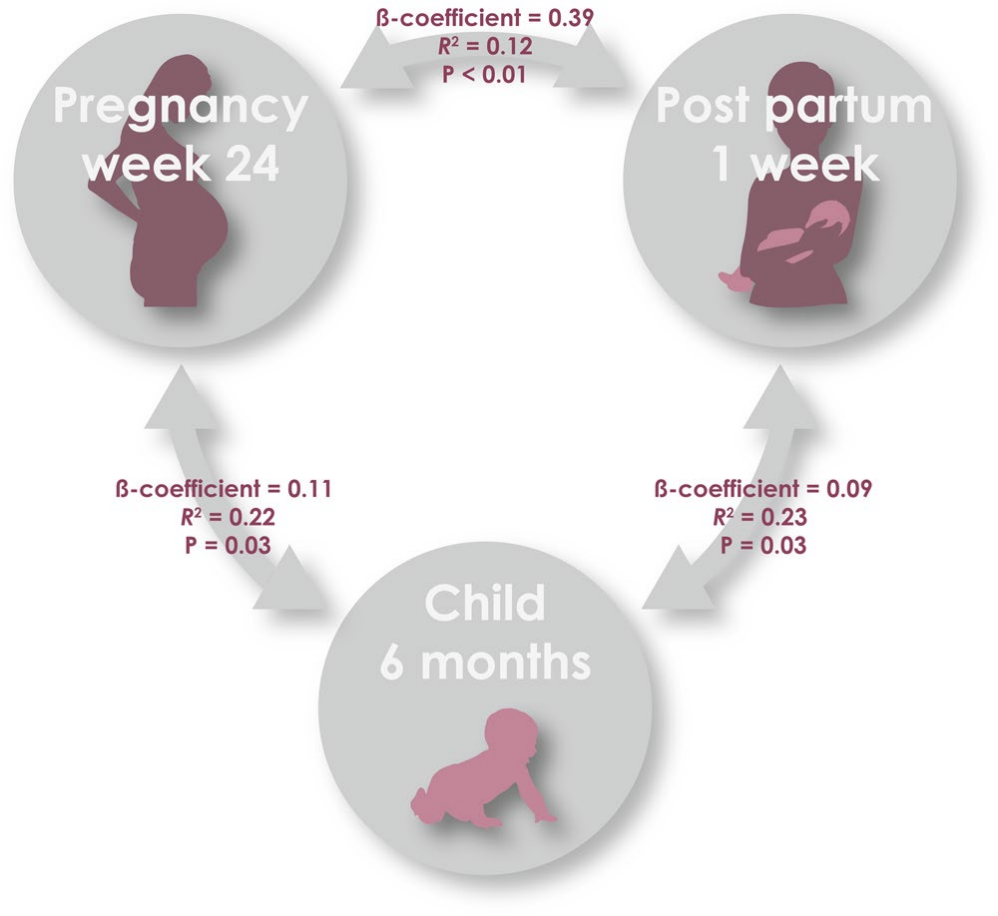
Correlation of systemic low-grade inflammation between mother and child, from pregnancy week 24, 1 week postpartum and child age 6 months.

The comparisons revealed significant associations between hs-CRP levels at all three time-points: mother week 24 of pregnancy vs. mother one-week postpartum, adjusted for social circumstances, season, BMI, delivery mode, antibiotics and smoking: β-coefficient = 0.39 [95% CI: 0.29–0.48], p < 0.01, *R*^2^ = 0.12; mother week 24 of pregnancy vs. child age 6 months, adjusted for infections, siblings, airway bacteria, social circumstances, season, CRP-GRS, antibiotics, smoking, child and mother BMI: β-coefficient = 0.11 [95% CI: 0.01–0.20], p = 0.03, *R*^2^ = 0.22; and mother one-week postpartum vs. child age 6 months, adjusted for infections, siblings, airway bacteria, season, delivery, CRP-GRS, child and mother BMI: β-coefficient = 0.09 [95% CI: 0.01–0.16], p = 0.03, *R*^2^ = 0.23), respectively (Fig. 2, Table 3).

**Table 3:** Multivariable association analysis of CRP levels with adjustment for all risk factors with p < 0.05, and multivariable association analysis of CRP with backward selection procedure.

	Est.	95% CI	R^2^	p-value
**Mother pregnancy week 24 vs. one-week postpartum**				
Crude analysis	0.37	[0.29–0.46]	0.09	<0.01
Multivariable analysis with all significant risk factors (p < 0.05)	0.39	[0.29–0.48]	0.12	<0.01
Multivariable analysis with backward selection	0.37	[0.27–0.45]	0.10	<0.01
**Mother pregnancy week 24 vs. child age 6 months**				
Crude analysis	0.10	[−0.01–0.16]	0.01	0.07
Multivariable analysis with all significant risk factors (p < 0.05)	0.11	[0.01–0.20]	0.22	0.03
Multivariable analysis with backward selection	0.11	[0.02–0.21]	0.21	0.02
**Mother one-week postpartum vs. child age 6 months**				
Crude analysis	0.08	[0.01–0.15]	0.01	0.03
Multivariable analysis with all significant risk factors (p < 0.05)	0.09	[0.01–0.16]	0.23	0.03
Multivariable analysis with backward selection	0.08	[0.01–0.16]	0.22	0.047

The above multivariable models including all significant risk factors from the univariate analyses compared to multivariable models including all risk factors with a backward selection procedure showed similar effect estimates and *R*^2^ values. Backward selection models of mother week 24 of pregnancy vs. one-week postpartum: β-coefficient = 0.37 [95% CI: 0.27–0.45], p < 0.01, *R*^2^ = 0.10; mother week 24 of pregnancy vs. child age 6 months: β-coefficient = 0.11 [95% CI: 0.02–0.21], p = 0.02, *R*^2^ = 0.21, and mother one-week postpartum vs. child age 6 months: β-coefficient = 0.08 [95% CI: 0.01–0.16], p = 0.047, *R*^2^ = 0.22 (Table 3).

Supplementary multivariable analyses were done on the associations between mother and child hs-CRP stratified for current infection, i.e. with and without current infections, showing a similar positive association in both strata (Supplementary Table 1). The correlation coefficients were largely the same in both strata suggesting that the covariates we adjusted the models for play a larger role for the size of the correlation coefficient than the higher hs-CRP levels in children with a history of infection.

Finally, to exclude that pleiotropic gene effects for hs-CRP and BMI could influence the findings we analyzed the association between child hs-CRP, the CRP-GRS and BMI. Increasing hs-CRP levels were associated with increasing CRP-GRS z-score: β-coefficient = 0.24 [95% CI: 0.14–0.34], p < 0.01, and with increasing BMI z-score: β-coefficient = 0.11 [95% CI: 0.01–0.21], p < 0.01. However, the CRP-GRS was not associated with BMI: β-coefficient = 0.03 [95% CI: −0.04–0.11], p = 0.35.

## Discussion

### Primary findings

Systemic low-grade inflammation assessed by hs-CRP levels were correlated during pregnancy and from pregnant women to their child. Higher hs-CRP levels during pregnancy and in early childhood were associated with higher mother and child BMI, cesarean section, smoking, infections, older siblings, bacterial airway colonization, a higher CRP genetic risk score, lower social circumstances and season of sampling. The associations between hs-CRP levels in the mother-child pairs were robust for adjustment for these risk factors, suggesting that intrauterine programming or additional unmeasured host, genetic or environmental factors might drive these mother-to-child hs-CRP correlations.

### Strengths and limitations

Previous studies have investigated risk factors of elevated hs-CRP in pregnant women^2^ and in children of varying ages^18^, but this is the first study providing a large dataset of pairwise assessments of hs-CRP in mothers and their children investigating whether low-grade systemic inflammation during pregnancy and early childhood are correlated.

The detailed, longitudinally collected information on anthropometrics, pregnancy complications, birth, and early childhood exposures is a significant strength of the study providing the foundation for robust confounder adjustments. All data were collected at the COPSAC research clinic following predefined standard operating procedures and doctor interviews based on questions with closed response categories, which ensure high quality assessments. Furthermore, whenever possible, data were validated against national register databases.

The detailed information on symptoms of infections captured by daily diary registrations and validated at visits to the COPSAC clinic^15^ is another significant advantage of the study as CRP is known to be elevated during and after infections^19^.

We calculated a CRP-genetic risk score based on the only available CRP meta-GWAS, which predominantly included data from adults^17^. This GRS appeared to be sensitive also in children as it was strongly associated with hs-CRP levels at age 6 months. However, the CRP-genetic risk score only explained part of the total variance of the hs-CRP levels; consequently, we cannot rule out that other genes may influence the CRP levels and the correlation between mother and child levels. Interestingly, the association between hs-CRP levels in mothers and their children persisted after adjusting for the genetic risk score, which argues against this association being solely genetically determined. Still, other genes may explain the observed correlation, particularly pleiotropic gene effects, e.g. shared risk variants for hs-CRP and BMI/overweight, which have shown to be coupled^20^. However, the CRP-genetic risk score was not associated with BMI in our data, which argues against a pleitropic gene effect.

The final multivariable regression models accounting for all potential confounders in our dataset revealed a consistent association between hs-CRP levels in mother and child with a marginal *R*^2^ up to 25%, which illustrates a good model fit, but also underscores that 75% of the variation remains unexplained.

### Interpretation

We found that degree of low-grade systemic inflammation determined by levels of hs-CRP in pregnant mothers and their children at age 6 months were correlated. The immune status of a pregnant woman is altered, which is mirrored in elevated CRP levels even during normal pregnancies^1,2^. Animal studies have not revealed any evidence of transplacental transport of CRP molecules to the fetus^21^, while human studies are lacking. The half-life of CRP is only 19 hours^22^. Therefore a direct transfer of CRP molecules is not a likely explanation for the observed correlation between maternal and child CRP levels.

There is however evidence that some immune cells, cytokines and chemokines may cross the placenta, with varying transplacental transfer in different stages of pregnancy, probably due to the fetus’ different needs during the different developmental phases^23^. The degree of inflammation and level of hs-CRP in the mother has been shown to regulate the flow of nutrients across placenta^23^, which may indirectly influence the immune status of the developing fetus, causing low-grade systemic inflammation. We observed correlation of hs-CRP levels from mother to child using both pregnancy week 24 and one-week postpartum assessments, which argues against a restriction of this phenomenon to specific pregnancy periods. Assessment of fathers hs-CRP level and investigating whether these were correlated with child levels would have provided important information on whether the observed association between mother and child levels were due to in utero programming of the fetus.

The validity of the hs-CRP assessments in the mothers is assured by reliable associations to several known risk factors such as maternal pre-pregnancy BMI above 25 kg/m^2^^23^. Moreover, antibiotic usage reflects a history of infections, which has been shown to result in elevated CRP for several weeks after the infection^19^, inflammation due to wound healing is well-known following cesarean section^22^, and smoking is known to cause airway and systemic inflammation^3^. Low social circumstances may be associated with a life-style more often including unhealthy dieting and proneness to develop inflammation^24^ and chronic inflammatory diseases^25^. Adjusting for these risk factors in the association analysis between hs-CRP at the maternal sampling points and between mother and child revealed even stronger associations and higher *R*^2^ values.

Even though the levels of hs-CRP increased dramatically from pregnancy week 24 to week 1 post partum, probably caused by giving birth, we still observed a specific hs-CRP signature of the individual mother, with e.g. maternal pre-pregnancy BMI as a common risk factor at both time points and a strong correlation between hs-CRP levels during pregnancy. The same was apparent for the correlation between mother and child hs-CRP levels even though the child’s hs-CRP levels were magnitudes lower. These observations point towards common effectors in mother and child.

We have previously demonstrated that neonatal bacterial airway colonization increases the risk of respiratory infections in early life^26^. Siblings in the household is moreover a well-known risk factor for infectious burden in the child^27^. We also found season to have a significant influence on the level of inflammation, which has also been observed in other studies^28^. We observed higher levels in mothers at pregnancy week 24 and child age 6 months during winter vs. summer, which may be due to an increased infectious load during winter, whereas the opposite was the case for hs-CRP levels at 1 week postpartum, which may be influenced by the recent childbirth. Finally, hs-CRP level was associated with the diary-verified history of current infections, which serves as a biological validation of the data.

Interestingly, we observed that higher BMI in both the mother and child was closely associated with higher level of hs-CRP. Overweight and obesity are known to be associated with systemic inflammation and hs-CRP levels in both children, adolescents and adults^20^, but an association between BMI in healthy infants and hs-CRP level has not been described previously. A few studies have shown that obesity in pregnancy is linked to risk of obesity and low-grade systemic inflammation in their children^5,6^, proposing this as the mechanism responsible for our observation of hs-CRP correlation from mother to child. However, adjusting the analysis for BMI still showed a significant association, indicating that this inflammatory trait is not exclusively related to BMI and obesity^29^.

This study investigates a wide range of environmental variables and finds strong correlations between hs-CRP level in mothers and their offspring independently of these factors. Still, it is a possibility that other environmental factors could account for additional effects on the level of hs-CRP and explain the correlation between mother and child levels. Our study demonstrates correlation of low-grade systemic inflammation from pregnant mothers to their children already by age 6 months. It can be speculated that this will influence the child’s risk of developing overweight and chronic inflammatory disorders later in childhood, but such cause-effect cannot be determined by our data.

## Conclusion

Systemic low-grade inflammation assessed by hs-CRP levels in pregnant mothers and their children are correlated independent of anthropometrics, environmental exposures during pregnancy and early childhood and CRP genetics. We speculate that this is suggestive of in utero programming of the fetus, which could have long-term health consequences for the child.

## Supplementary information

Supplementary table and figure

## Data Availability

The datasets generated during and/or analysed during the current study are available from the corresponding author on reasonable request.
