# Supplementary material for: Levels of Systemic Low-grade Inflammation in Pregnant Mothers and Their Offspring are Correlated
Source: Sci Rep. 2019 Feb 28;9:3043. doi: 10.1038/s41598-019-39620-5 (PMC6395736; doi:10.1038/s41598-019-39620-5)

Title page; Supplementary Information

Levels of Systemic Low-grade Inflammation in Pregnant Mothers and Their Offspring are Correlated

Authors: Nadia Rahman Fink, MD[1], Bo Chawes, MD, PhD, DMSc[1], Klaus Bønnelykke[1], MD, PhD, Jonathan Thorsen[1], MD, Jakob Stokholm, MD, PhD[1+2], Morten Arendt Rasmussen[1+3], MSc, PhD, Susanne Brix[4], MSc, PhD, Hans Bisgaard, MD, DMSc[1].

Affiliation:

1) COPSAC, Copenhagen Prospective Studies on Asthma in Childhood, Copenhagen University Hospital, Herlev-Gentofte,, Denmark
2) Department of Pediatrics, Naestved Hospital, Naestved, Denmark.
3) Department of Food Science, Faculty of Science, University of Copenhagen, Copenhagen, Denmark
4) Department of Biotechnology and Biomedicine, Technical University of Denmark, Lyngby, Denmark.

Correspondence:

Professor Hans Bisgaard, MD, DMSc

E-mail: bisgaard@copsac.com

Website: www.copsac.com

Supplement table 1. Associations between mother and child systemic low-grade inflammation, with hs-CRP levels stratified for current infections (within 14 days) at time of blood sampling.

	**Children *with* infections (n= 175)**				**Children *without* infection (n= 384)**				**All children (n = 640)**			
	**Est.**	**95% CI**	**R^2**	**p**	**Est.**	**95% CI**	**R^2**	**p**	**Est.**	**95% CI**	**R^2**	**p**
**Mother pregnancy week 24 vs. offspring age 6 months**												
Multivariable analysis with all significant risk factors (p<0.05)	0.09	[-0.09 – 0.27]	0.10	0.32	0.10	[-0.02 – 0.21]	0.18	0.11	0.11	[0.01–0.20]	0.22	0.03
Backward selected model	0.06	[-0.08 – 0.20]	0.08	0.38	0.06	[-0.04 – 0.17]	0.17	0.25	0.11	[0.02–0.21]	0.21	0.02
**Mother one-week postpartum vs. offspring age 6 months**												
Multivariable analysis with all significant risk factors (p<0.05)	0.08	[-0.05 – 0.22]	0.10	0.23	0.09	[-0.01 – 0.19]	0.20	0.05	0.09	[0.01–0.16]	0.23	0.03
Backward selected model	0.10	[-0.02 – 0.22]	0.09	0.09	0.07	[-0.02 – 0.16]	0.18	0.13	0.08	[0.02–0.21]	0.22	0.047

Supplement figure 1a-c. CRP risk factor analysis for the three time-points: pregnancy week 24 (panel A), one-week postpartum (panel B) and children age 6 months (panel C). The figures depict geometric mean ratio (95% CI).

a) Pregnancy week 24

b) One-week postpartum

c) Children age 6 month

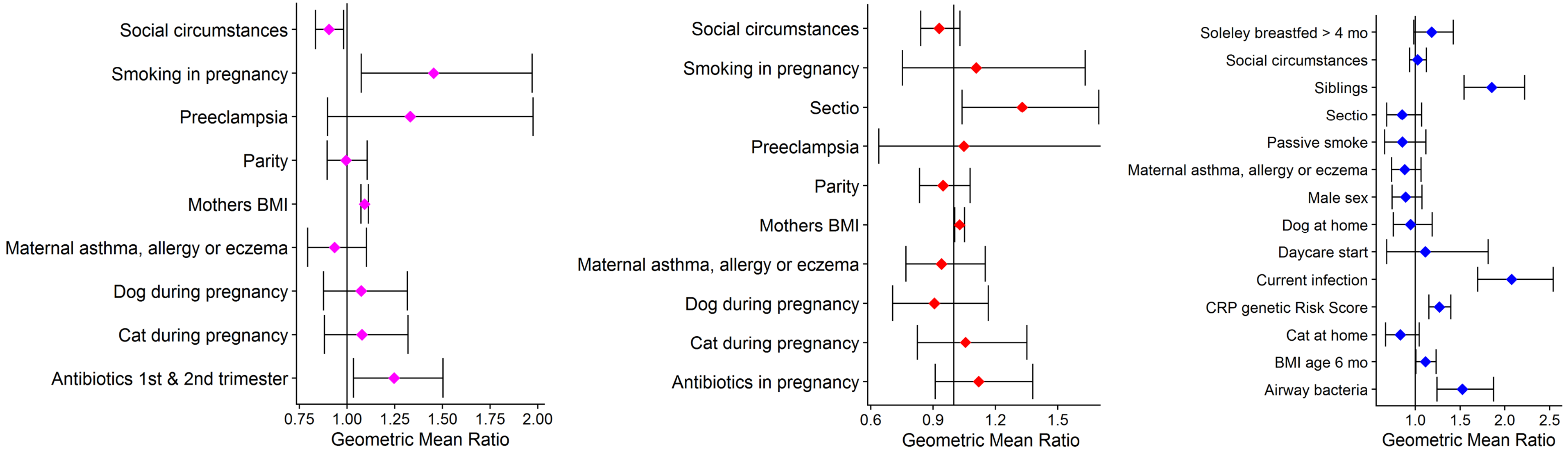

Supplement: Supplementary file 1 — Supplementary table and figure [file 41598_2019_39620_MOESM1_ESM.pdf]
